# Measurement of spinal cord atrophy using phase sensitive inversion recovery (PSIR) imaging in motor neuron disease

**DOI:** 10.1371/journal.pone.0208255

**Published:** 2018-11-29

**Authors:** Nicholas T. Olney, Antje Bischof, Howard Rosen, Eduardo Caverzasi, William A. Stern, Catherine Lomen-Hoerth, Bruce L. Miller, Roland G. Henry, Nico Papinutto

**Affiliations:** 1 Department of Neurology, Memory and Aging Center, University of California San Francisco, San Francisco, California, United States of America; 2 Department of Neurology, University of California San Francisco Amyotrophic Lateral Sclerosis Center, University of California San Francisco, San Francisco, California, United States of America; 3 Department of Neurology, University of California San Francisco, San Francisco, California, United States of America; 4 Department of Neurology and Immunology Clinic, Departments of Medicine, Biomedicine and Clinical Research, University Hospital Basel, Basel, Switzerland; Charite Universitatsmedizin Berlin, GERMANY

## Abstract

**Background:**

The spectrum of motor neuron disease (MND) includes numerous phenotypes with various life expectancies. The degree of upper and lower motor neuron involvement can impact prognosis. Phase sensitive inversion recovery (PSIR) imaging has been shown to detect *in vivo* gray matter (GM) and white matter (WM) atrophy in the spinal cord of other patient populations but has not been explored in MND.

**Methods:**

In this study, total cord, WM and GM areas of ten patients with a diagnosis within the MND spectrum were compared to those of ten healthy controls (HC). Patients’ diagnosis included amyotrophic lateral sclerosis (ALS), primary lateral sclerosis, primary muscular atrophy, facial onset sensory and motor neuronopathy and ALS-Frontotemporal dementia. Axial 2D PSIR images were acquired at four cervical disc levels (C2-C3, C3-C4, C5-C6 and C7-T1) with a short acquisition time (2 minutes) protocol. Total cross-sectional areas (TCA), GM and WM areas were measured using a combination of highly reliable manual and semi-automated methods. Cord areas in MND patients were compared with HC using linear regression analyses adjusted for age and sex. Correlation of WM and GM areas in MND patients was explored to gain insights into underlying atrophy patterns.

**Results:**

MND patients as a group had significantly smaller cervical cord GM area compared to HC at all four levels (C2-C3: p = .009; C3-C4: p = .001; C5-C6: p = .006; C7-T1: p = .002). WM area at C5-C6 level was significantly smaller (p = .001). TCA was significantly smaller at C3-C4 (p = .018) and C5-C6 (p = .002). No significant GM and WM atrophy was detected in the two patients with predominantly bulbar phenotype. Concomitant GM and WM atrophy was detected in solely upper or lower motor neuron level phenotypes. There was a significant correlation between GM and WM areas at all four levels in this diverse population of MND.

**Conclusion:**

Spinal cord GM and WM atrophy can be detected *in vivo* in patients within the MND spectrum using a short acquisition time 2D PSIR imaging protocol. PSIR imaging shows promise as a method for quantifying spinal cord involvement and thus may be useful for diagnosis, prognosis and for monitoring disease progression.

## Introduction

The distinct spinal cord pathology of amyotrophic lateral sclerosis (ALS) was first described by Charcot over 100 years ago [1, 2]. ALS is now considered part of a larger heterogeneous spectrum of motor neuron diseases (MND) [3], that includes (but is not limited to): ALS, primary lateral sclerosis (PLS), progressive muscular atrophy (PMA), ALS and frontotemporal dementia (ALS-FTD), and facial-onset sensory and motor neuronopathy (FOSMN) [3–5]. These four MND phenotypes have recently been associated with TDP-43 (transactive response DNA-binding protein 43) neuropathology found on autopsy, and it has been posited that they are linked as TDP-43 proteinopathies [2, 5]. It is currently unclear, whether these phenotypes represent a continuum rather than distinct entities. In addition, clinical differentiation between MND subtypes remains challenging [6–9]. Importantly, prognosis differs between and even within these phenotypes, with an average survival of three to five years from onset in ALS compared to a slower disease progression in PMA or PLS [10, 11]. Therefore, novel measures to better assess and characterize these phenotypes are an unmet need [12].

MRI has been proposed to provide *in vivo* biomarkers to assess central nervous system abnormalities in ALS [12–14]. Extensive work has established the utility of brain imaging, mostly focusing on the detection of atrophy, and microscopic abnormalities with diffusion-weighted techniques [12, 15–23]. However, most of the quantitative studies reported results at a group level, raising the question of the applicability for single patients in the clinical setting [17]. The investigation of spinal cord (SC) changes has been hampered by technical challenges [24].

Conventional SC MRI protocols have poor gray matter (GM) and white matter (WM) contrast, insufficient to measure changes related to UMN and LMN dysfunction [25]. Recent work suggests that the assessment of spinal cord GM and WM from T2*-weighted images is feasible in ALS patients and correlates with clinical disability [26, 27]. However, T2*-weighted imaging can be technically challenging at lower cervical cord levels, as it is strongly affected by susceptibility and by movement, predominantly from respiration, swallowing, heartbeat and CSF pulsation [24].

Recently, a novel imaging technique based on phase sensitive inversion recovery (PSIR) has been implemented for spinal cord [28]. PSIR was originally developed in cardiac imaging [29] with low sensitivity to motion and susceptibility and offers an enhanced T1-weigthing, ideal for total cord segmentation [30]. It has already been demonstrated that the short acquisition time 2D PSIR protocol can easily be added to clinical MRI protocols for quantification of GM and WM tissues in the spinal cord of healthy controls (HC) [28, 31]. In multiple sclerosis, PSIR has been successfully applied to a large cohort of patients and has been shown to reliably detect WM and GM atrophy that correlates with clinical disability [32, 33].

The aim of this cross-sectional study was to determine the potential of PSIR imaging to detect WM and GM atrophy in MND, and to determine whether cervical cord atrophy patterns correlate with clinical phenotypes or provide additional information on subclinical structural involvement. In order to assess the relationship between PSIR-based WM and GM measurements and clinical presentation, we studied a heterogeneous group of patients with various patterns of upper and lower motor neuron involvement at multiple cervical cord levels.

## Methods

### Research participants

Ten patients with a diagnosis within the MND spectrum [3, 4] were recruited from the UCSF ALS clinic for PSIR imaging between December 2016 and August 2017. MND had been diagnosed by clinical and electrophysiological examination at the discretion of the treating physician at the UCSF ALS clinic. The revised ALS functional rating scale (ALSFRS-R) had been obtained by a neurologist for all patients [34, 35]. Clinical data of diagnosis, neurologic examination and ALSFRS-R were obtained via chart review. PSIR images acquired on 10 HC to measure WM and GM areas of the whole spinal cord and related data described in previous studies (S1 Table) were used for comparison with findings in the MND group [28, 31].

The Institutional Review Board (IRB) at University of California San Francisco (UCSF) approved the study protocols. Written informed consent was obtained from all participants.

### Image acquisition

**Fig 1:**
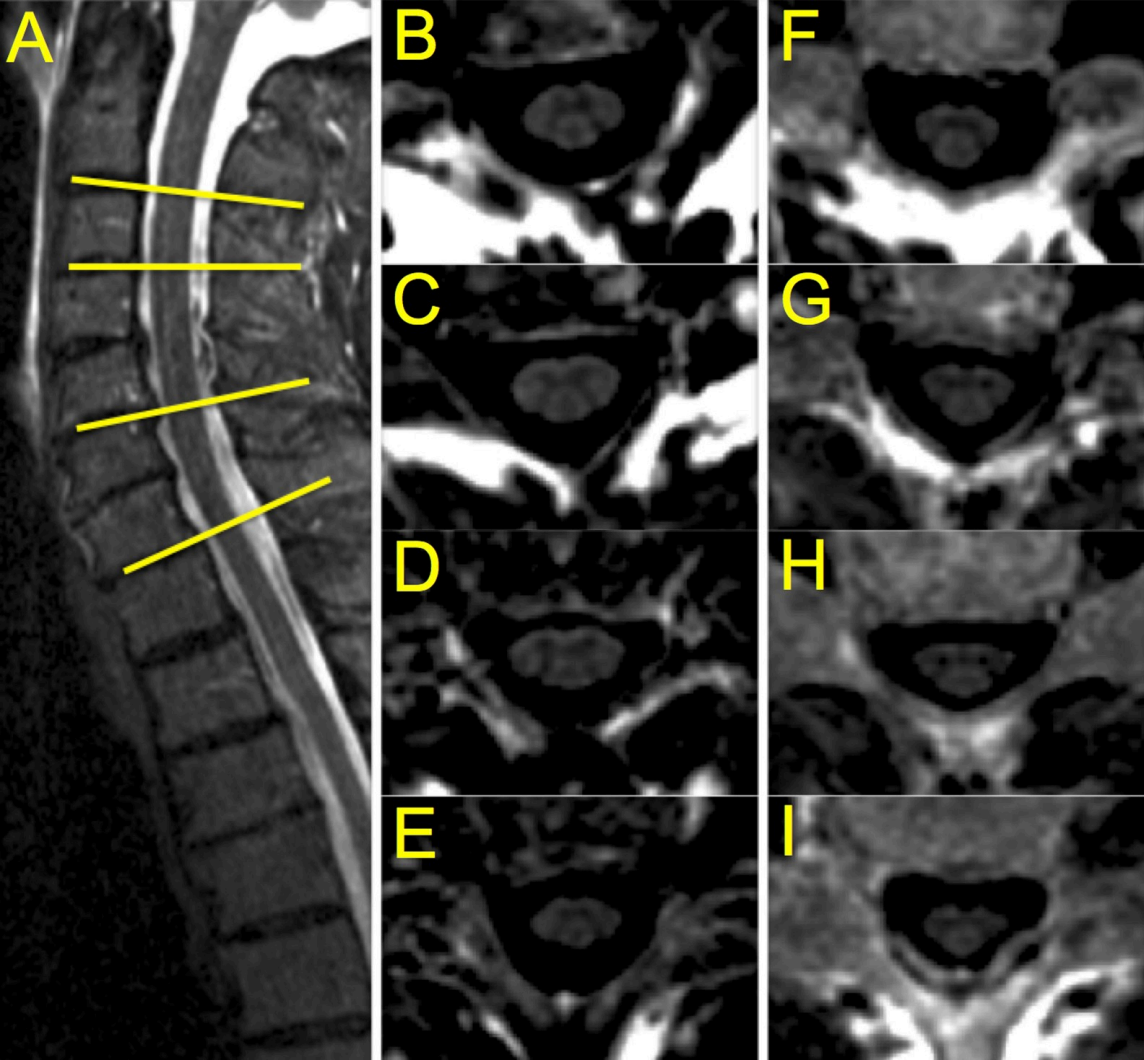
Illustration of PSIR acquisition on two motor neuron disease patients. A) Sagittal short tau inversion recovery (STIR) image used to prescribe the PSIR acquisitions at the four cervical disc levels (yellow lines). B-E) PSIR acquisitions (phase-sensitive reconstructed images are shown) of a patient with predominantly bulbar symptoms at C2-C3, C3-C4, C5-C6 and C7-T1 disc level, respectively; F-I) PSIR acquisitions of the patient with PLS at C2-C3, C3-C4, C5-C6 and C7-T1 disc level, respectively. Notice how gray and white matter are visibly reduced at all four levels in the PLS patient when compared with the patient with bulbar involvement.

All participants underwent MRI using a Siemens 3T Skyra scanner. A 64-channel head-neck coil, providing good signal-to-noise ratio within the upper cervical cord, was used. For the lowest cervical levels, the spinal coil arrays in the scanner bed were used. Single slice axial 2D PSIR images were acquired at four cervical intervertebral disc levels: C2-C3, C3-C4, C5-C6 and C7-T1. The 2D PSIR acquisitions were prescribed on a standard sagittal short tau inversion recovery (STIR) image using the vertebral disc as reference and positioning the slices perpendicular to the SC (Fig 1).

The 2D PSIR protocol was set up for an optimized GM/WM contrast to noise ratio[28] using the following key parameters: axial in plane resolution 0.78x0.78 mm^2^, slice thickness 5 mm, matrix 256x256, TR/TE/TI = 4000/3.22/400 ms, angle = 10°, number of averages: 3, acquisition time: 1:50 min, magnitude and phase-sensitive reconstructed images. The short acquisition time allowed repeating a PSIR acquisition. MRI technologists were instructed to repeat the acquisitions if they had doubts regarding image quality and if time allowed. If multiple images were acquired, the best scan for each patient and level was selected for analysis following consensual decision of three operators with more than 10 years of experience in neuroimaging (AB, EC and NP).

### Image analysis

Total cross-sectional area (TCA) and GM areas of the participants were measured on the phase-sensitive reconstructed images. TCA estimates were obtained in a semi-automated way by a single blinded experienced operator (NP) using an active surface model [36] (using the software JIM, version 6, Xinapse Systems, http://www.xinapse.com) with a method previously shown to have high intra- and inter-rater reliability [28, 32]; briefly this was done using the *cord finder toolkit* with fixed settings (nominal cord diameter 8mm, number of shape coefficients 24, order of longitudinal variation 12). The marker requested by the toolkit was positioned on the mid-sagittal WM, directly posterior to the gray commissure.

GM areas were manually measured using JIM with a segmentation technique that has been shown to be highly reliable [28, 32]. GM automated segmentation techniques have recently been implemented in our post-processing pipeline [37]. Nevertheless, all available automated methods have been developed using HC datasets [38], and their reliability has not been exhaustively tested on pathological SC images. We therefore preferred to perform manual GM segmentation since we believe that automated segmentation could bias the results, especially in MND, where previous data are not available and the GM atrophy is expected to be regionally dependent.

Three operators (AB, EC and NP) were blinded to clinical diagnosis and manually segmented the GM area three times using JIM after having trained on a subset of images acquired on the 10 HC [28, 31]. Specifically, the C3-C4 and C7-T1 levels were chosen. For each participant and level, the average GM area obtained from the three segmentations was calculated. The average measure across the three operators was finally computed to reduce inter-rater variability, and used for further analyses. The WM area for each subject was calculated at each intervertebral disc level as the difference between the TCA and the average GM area at that level.

### Statistical analysis

Statistical analysis was performed using JMP Pro 13 and Stata version 14.1. The significance level was set at α = 0.05 for all analyses.

#### Reliability assessments

To test for inter-rater reliability, intra-class correlation coefficients (ICC) of average GM areas were computed among the values calculated by three operators from the HC training dataset and the previously reported values (two-way random-effects model for the mean of raters [39]). ICC were computed for the GM area of the MND patients.

#### Comparison of cord areas between controls and MND patients

Linear regression analyses were used to compare PSIR derived areas (TCA, GM, WM) at each level between MND patients and the 10 HC data available from previous work used here as normative reference, adjusting for age and sex.

To statistically describe the extent of atrophy, Z scores were created for PSIR derived areas of GM and WM for each level examined in each patient using the mean and standard deviation of the 10 HCs. To create a single descriptive value per patient, total average Z scores of GM and WM were created per patient by adding the Z scores at each level and dividing by the number of levels examined.

In order to compare the level of abnormalities across MND patients with different clinical presentations, Pearson *r* correlation coefficients between GM and WM areas were computed at each examined spinal cord level. For comparison, this was done also for HCs.

#### Correlations of MRI metrics with clinical syndrome and disability

A disease progression rate (DPR) was calculated as described previously[35] using the ALSFRS-R scores and the following formula: (48—ALSFRS-R)/disease duration in months. In addition we also created an arm DPR because our PSIR sequence focused on the cervical regions. This was accomplished by focusing on the two ALSFRS-R questions that assess upper extremities (UE) symptoms (questions 4 and 5, which assess changes in handwriting and cutting food, respectively) and dividing by disease duration in months (8—UE ALSFRS-R)/disease duration in months.

Linear regression analyses were performed to examine whether the GM and WM areas predicted total ALSFRS-R, total DPR, and arm DPR.

## Results

### Participant demographics

Ten patients with a diagnosis within the MND spectrum were included with mean age of 58.7 years (SD = 11.4). The MND cohort consisted of patients with ALS (2), FOSMN (2), PLS (1), PMA (2), FTD-ALS (2), and bulbar-onset MND (1). Their clinical characteristics are reported in Tables 1, 2 and 3. Of note, two patients (#8 and #10) had only bulbar motor symptoms with no limb involvement. Three patients (#3, #5 and #7) had both upper and lower motor neuron signs, 2 of these meeting criteria for “typical” ALS [40], and another with FOSMN. The patient #6 that had solely UMN signs on exam and negative EMG would meet clinical diagnostic criteria for PLS [8]. Four patients (#1, #2, #4 and #9) had clinical exams consistent with LMN predominant MND phenotypes. The patients with frontotemporal dementia (#8 and #9) meet criteria for probable FTD using Rascovsky 2011 criteria [41]. Mean age of the 10 HC (5 females), whose SC acquisitions and areas were available at all four cervical disc levels, was 37.7 years (SD = 7.5).

**Table 1:** Clinical characteristics and white and gray matter Z scores of patients with motor neuron disease.

#	Sex	Age	Dx	Motor phenotype	Onset	ALSFRS-R	DD	Total DPR	UE DPR	Z GM	Z WM
1	M	64	PMA	LMN	Arm	40	29	0.28	0.21	-1.57	-2.04
2	F	62	FOSMN	LMN	Arm	27	28	0.75	0.18	-2.47	-0.34
3	M	74	ALS	UMN+LMN	Arm	33	28	0.54	0.14	-2.33	-2.08
4	M	33	PMA	LMN	Arm	27	36	0.58	0.19	-3.23	-3.99
5	M	52	FOSMN	UMN+LMN	Arm	31	60	0.28	0.03	-4.41	-5.62
6	M	58	PLS	UMN	Leg	42	128	0.05	0.01	-2.39	-3.49
7	M	59	ALS	UMN+LMN	Arm	36	48	0.25	0.04	-2.10	-0.22
8	M	72	FTD-ALS	Bulbar UMN+LMN	Bulbar	41	51	0.14	0	-0.47	0.48
9	M	56	FTD-ALS	LMN	Bulbar	44	11	0.36	0.09	-1.40	-2.28
10	F	57	ALS	Bulbar UMN+LMN	Bulbar	33	21	0.71	0	-0.38	0.74

#: patient number; F: Female; M: Male; Dx: motor neuron disease diagnosis; Motor phenotype: degree of upper motor neuron (UMN) and lower motor neuron (LMN) signs detected in clinical exam (for more details refer to Table 3); Onset: site of motor symptom onset; ALSFRS-R: total score; DD: disease duration expressed in months; Total DPR: total disease progression rate; UE DPR: upper Extremity disease progression rate; Z GM: total Z scores of GM; Z WM: total Z scores of WM.

**Table 2:** Involved topographical anatomic regions and symptom severity in motor neuron disease patients.

#	Dx	Regions involved	ALSFRS-R	Bulbar	UE	LE
1	PMA	UE only	40	12	2	8
2	FOSMN	Bulbar>UE	27	4	3	8
3	ALS	LE>UE>Bulbar	33	10	4	3
4	PMA	UE>LE>Bulbar	27	11	1	3
5	FOSMN	Bulbar>UE>LE	31	6	6	7
6	PLS	LE>UE>Bulbar	42	11	7	5
7	ALS	LE>UE>Bulbar	36	9	6	5
8	FTD-ALS	Bulbar only	41	5	8	8
9	FTD-ALS	Bulbar>UE	44	9	7	8
10	ALS	Bulbar only	33	0	8	8

#: patient number; Dx: motor neuron disease diagnosis; Regions involved: summary of regions involved and severity as reported by ALSFRS-R rating scale scores; ALSFRS-R: total score; Bulbar: sum of ALSFRS-R questions 1, 2 and 3 relating to bulbar symptoms (speech, salivation, swallowing–maximum score: 12-); UE: sum of ALSFRS-R questions 4 and 5 relating to upper extremity symptoms (handwriting and cutting food–maximum score: 8-); LE: sum of questions 8 and 9 that relate to lower extremity symptoms (walking and climbing stairs–maximum score: 8-).

**Table 3:** Cognitive and motor findings of patients with motor neuron disease.

#	Dx	Motor phenotype	Cognitive	Jaw Jerk	Reflexes	Tone	UE Strength	LE strength	EMG
1	PMA	LMN	-	-	↓	↓	2/2	5/5	+
2	FOSMN	LMN	-	-	Nml	↓	4/2	5/5	+
3	ALS	UMN+LMN	-	+	↑	↑	4/2	4/4	+
4	PMA	LMN	-	NA	↓	↓	0/0	4/4	+
5	FOSMN	UMN+LMN	-	-	Nml	↑	4/4	5/5	+
6	PLS	UMN	-	-	↑	↑	5/4	5/5	-
7	ALS	UMN+LMN	-	+	↑	↑	4/4	4/4	+
8	FTD-ALS	Bulbar UMN+LMN	+	+	Nml	Nml	5/5	5/5	+
9	FTD-ALS	LMN	+	+	Nml	Nml	5/5	5/5	+
10	ALS	Bulbar UMN+LMN	-	+	Nml	Nml	5/5	5/5	+

If a clinical symptom is supportive of an UMN or LMN it is noted in parenthesis in the below description.

Abbreviations

#: patient number; Dx: motor neuron disease diagnosis; Motor phenotype: degree of upper motor neuron (UMN) and lower motor neuron (LMN) signs detected in clinical exam; Cognitive: “+” cognitive changes, “-”no cognitive changes; Jaw Jerk: “+” present (UMN), “-”absent, “NA” not available; Reflexes: summary of reflexes increased (↑, UMN), decreased (↓, LMN) or normal (Nml); Tone: increased (↑, UMN), decreased (↓, LMN), normal (Nml); UE/LE strength: upper/lower extremity strength: most commonly reported number (mode) recorded per limb (Right/Left); EMG: “+” electrophysiological evidence of LMN involvement, “-”no LMN involvement.

### Quality control/ Inter-rater reliability assessment

Spinal cord PSIR images of the HC previously published did not show any abnormalities. On HC, the MRI technologists never had to repeat an acquisition due to suboptimal quality.

The inter-rater reliability among all GM area measurements performed by the three operators in the HC training dataset and the published data demonstrated an ICC of 0.9856. These results on the HC training data subset support the use of previously published data as normative reference for the present study.

The SC scans of the 10 MND patients did not show evidence of other disease processes that contradict the clinical diagnoses. On 3 patients there was no need of repeating any acquisition, while for 7 of them at least one acquisition at one level was repeated by the MRI technologists. The average number of times PSIR images were acquired (for all patients and all levels) was 1.6. Despite the repetition of PSIR acquisition for poor quality images, C5-C6 level acquisitions were consensually judged to be of insufficient quality by AB, EC and NP in two patients and eliminated from the analysis. The ICC for the GM segmentations of the three operators on MND patients was 0.9173, confirming a high inter-rater reliability of the segmentation method on MND patients.

### Comparison of cord areas between controls and MND patients

**Fig 2:**
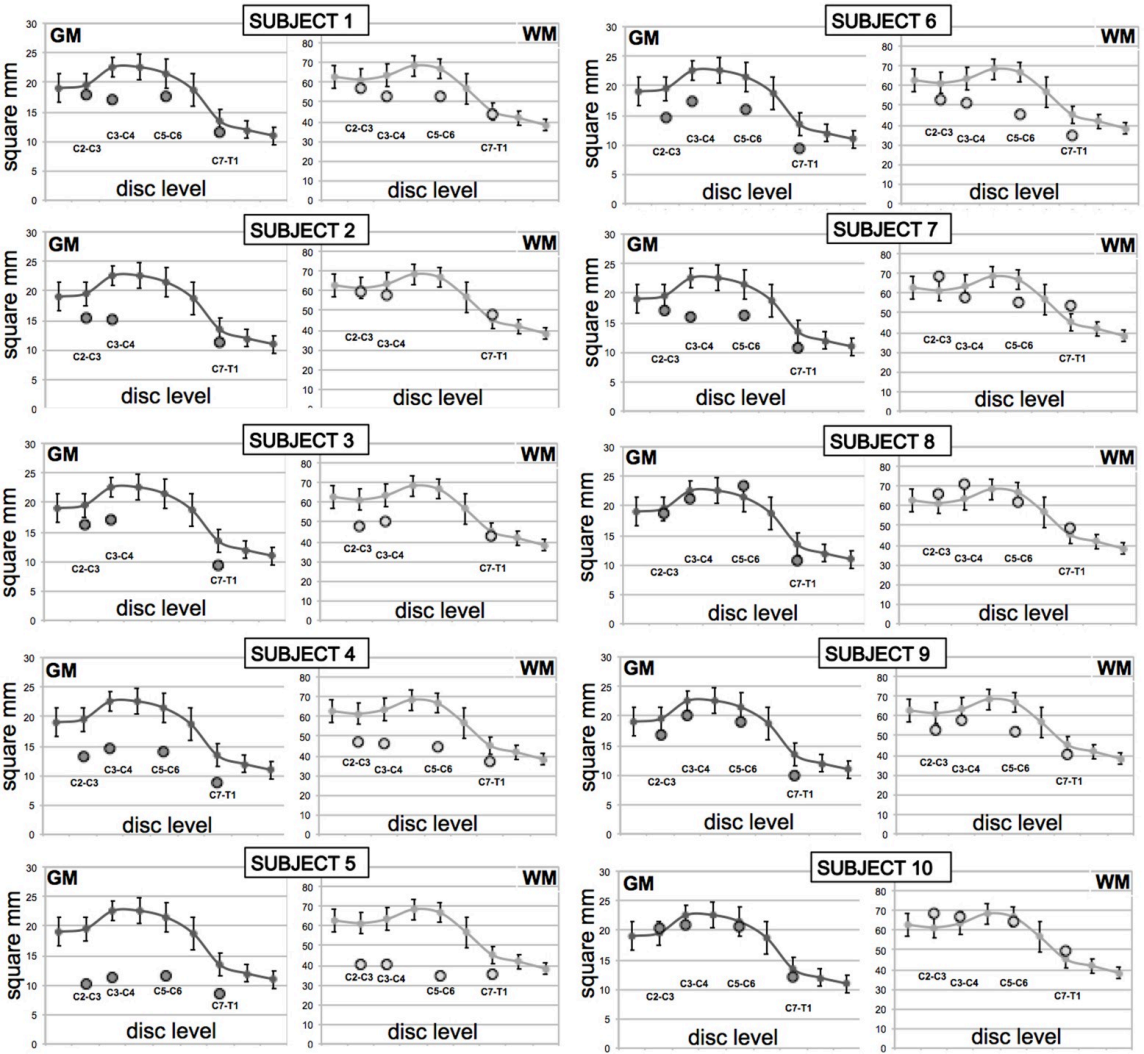
Spinal cord gray (GM) and white matter (WM) areas of individual motor neuron disease (MND) patients. GM and WM areas of the 10 MND patients (subject 1–10) at the four cervical levels (circles) compared to 10 healthy controls (solid line, data from Papinutto 2015[28]). Whiskers represent standard deviations.

GM and WM areas were individually plotted for each patient compared to controls as shown in Fig 2 with all values in Table 4.

**Table 4:** Individual spinal cord gray (GM) and white matter (WM) areas of the 10 motor neuron disease (MND) patients.

Patient #	C2-C3 GM	C3-C4 GM	C5-C6 GM	C7-T1 GM	Z GM	C2-C3 WM	C3-C4 WM	C5-C6 WM	C7-T1 WM	Z WM
HC	19.54 (2.07)	22.59 (1.70)	21.49 (2.58)	13.40 (1.93)	-	61.55 (4.72)	63.60 (4.65)	66.82 (3.18)	45.23 (3.74)	-
1	18.03	17.13	17.72	11.65	-1.57	56.90	52.53	52.71	43.90	-2.04
2	15.41	15.12	-	11.38	-2.47	59.35	57.55	-	48.01	-0.34
3	16.17	17.14	-	9.24	-2.33	48.14	50.20	-	43.33	-2.08
4	13.20	14.68	14.09	8.85	-3.23	47.08	46.59	44.58	36.74	-3.99
5	10.07	11.17	11.47	8.63	-4.41	40.13	40.28	34.26	35.17	-5.62
6	14.73	17.43	16.11	9.27	-2.39	52.71	51.25	45.58	34.87	-3.49
7	17.19	16.05	16.25	10.68	-2.10	68.72	58.15	55.59	53.89	-0.22
8	18.61	21.21	23.34	10.80	-0.47	66.26	70.84	62.04	48.54	0.48
9	16.71	20.11	18.93	9.93	-1.40	52.97	57.57	51.72	40.46	-2.28
10	20.33	20.99	20.56	12.21	-0.38	69.00	67.27	64.86	49.73	0.74

GM area and WM area for the 10 MND patients in square mm. The first line (HC) is the mean value/standard deviation for the 10 HC. For patients 1–10 the individual values at each level are listed. The total Z score (average Z score across the levels) for GM and WM are also reported.

The patients with bulbar predominant motor symptoms (#8 and #10) did not show atrophy when compared to controls (Fig 2, Table 4). It is also worth noting that the patient with pure UMN phenotype (#6) showed atrophy in the GM, while only WM atrophy would be expected. Conversely 3 of the 4 patients with predominantly LMN clinical phenotypes (#1, #4 and #9), where only GM atrophy would be predicted, did show atrophy in both GM and WM.

**Fig 3:**
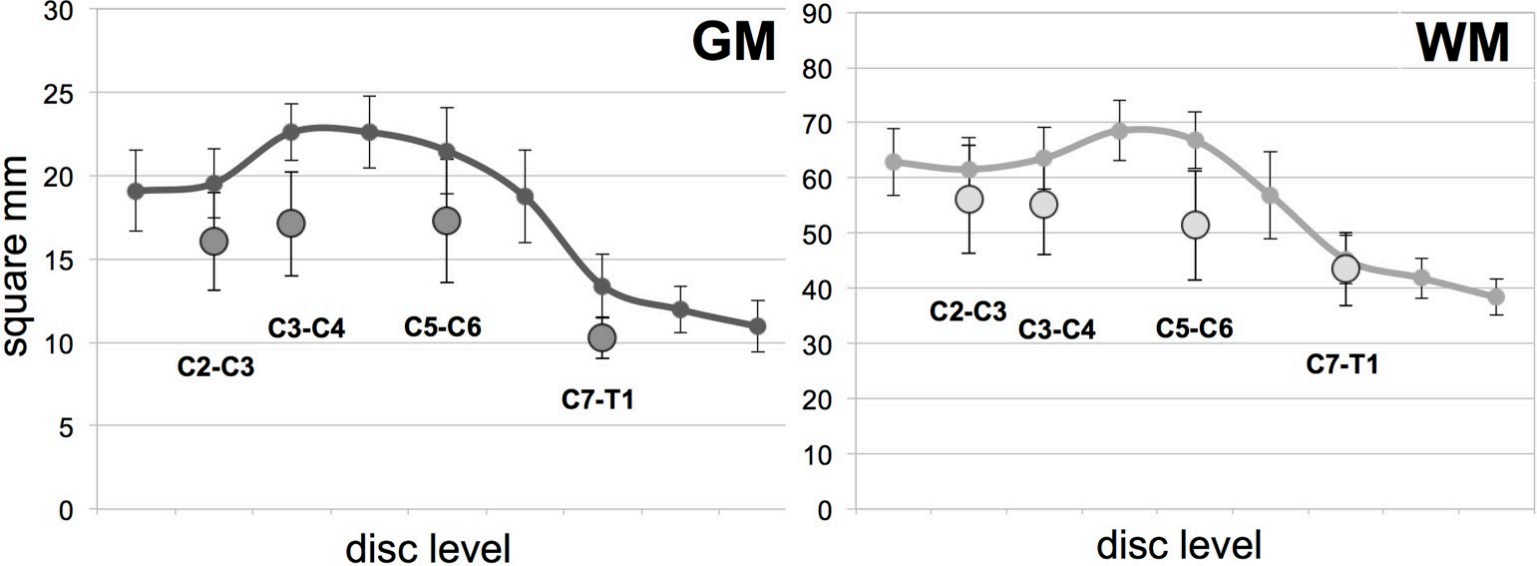
Spinal cord gray (GM) and white matter (WM) areas of all 10 motor neuron disease (MND) patients and healthy controls. Average GM (left) and WM area (right) of the 10 MND patients at the four cervical levels (circles) compared to 10 healthy controls (solid line, data from Papinutto 2015[28]). Whiskers represent standard deviations.

Group average GM and WM areas of the MND patients versus healthy controls are illustrated in Fig 3 and reported in Table 5.

**Table 5:** Comparison of PSIR measurements for total cord, spinal cord gray and white matter areas (mm^2^) between healthy controls and motor neuron disease patients (MND).

Disc Level	Area	Controls Adjusted mean mm^2^ (SE)	MND Adjusted mean mm^2^ (SE)		Difference Between Means, Mean mm^2^ (SE)	Difference Between Means, 95% CI	P value
C2-C3	TCA	83.03 (4.06)	70.88 (4.28)		-12.15 (6.90)	-26.78 to 2.48	.098
	WM	62.73 (3.24)	55.79 (3.41)		-6.93 (5.50)	-18.6 to 4.70	.225
	GM	20.30 (1.04)	15.09 (1.10)		-5.22 (1.76)	-8.96 to -1.48	**.009**
C3-C4	TCA	88.29 (3.93)	70.75 (4.15)		-17.54 (6.68)	-31.70 to -3.38	**.018**
	WM	65.14 (3.04)	54.45 (3.20)		-10.69 (5.16)	-21.63 to 0.24	.055
	GM	23.15 (1.05)	16.30 (1.10)		-6.85 (1.78)	-10.61 to -3.09	**.001**
C5-C6	TCA	91.48 (3.71)	65.26 (4.67)		-26.22 (6.81)	-40.83 to -11.61	**.002**
	WM	69.07 (2.64)	49.76 (3.32)		-19.31 (4.85)	-29.72 to -8.91	**.001**
	GM	22.41(1.17)	15.50 (1.47)		-6.91 (2.14)	-11.50 to -2.31	**.006**
C7-T1	TCA	60.61 (2.56)	51.98 (2.70)		-8.63 (4.35)	-17.85 to 0.59	.065
	WM	46.69 (2.23)	42.32 (2.36)		-4.37 (3.79)	-12.42 to 3.67	.266
	GM	13.91 (0.67)	9.66 (0.71)		-4.25 (1.14)	-6.66 to -1.84	**.002**

Ordinary least squares linear regression analysis was performed with age and sex as covariates. Significant corrected p values (2-tailed) are reported in bold. C: cervical; T: thoracic; TCA: total cord area; WM: white matter; GM: gray matter; SE: standard error; CI: confidence interval; PSIR: phase sensitive inversion recovery.

Linear regression analyses adjusting for age and sex showed statistically significant differences when comparing TCA, GM and WM areas between HC and MND (Table 5). GM areas in MND patients were significantly smaller than in HC at all examined levels. WM area was significantly smaller in MND patients at C5-C6 level. TCA was significantly smaller in MND patients at C3-C4 and C5-C6 levels.

It is worth noting that these differences were statistically significant even when including the 2 patients with bulbar predominant symptoms with relatively normal GM and WM areas.

**Fig 4:**
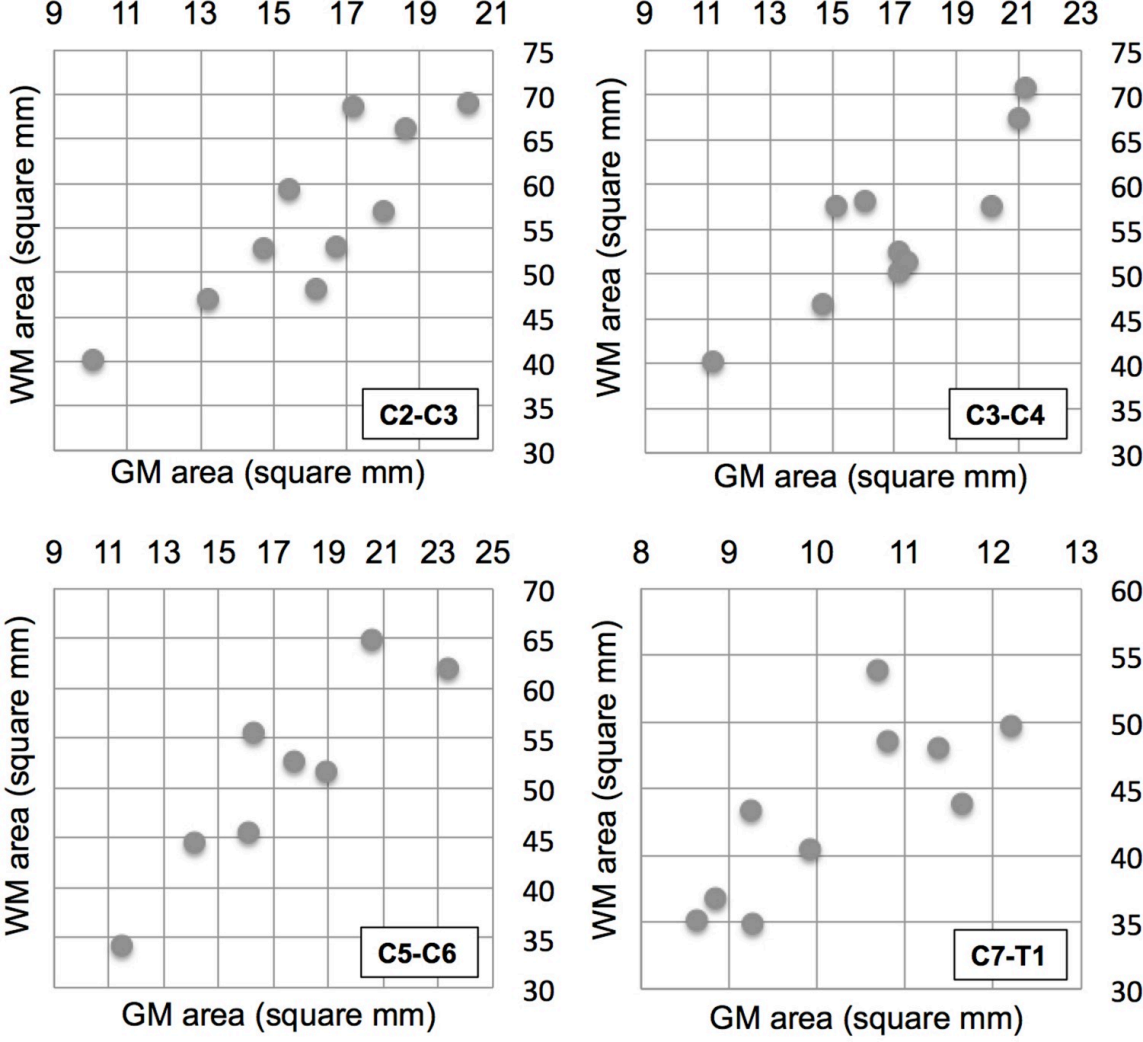
Association of gray and white matter spinal cord atrophy. Gray matter area (x-axis) and white matter area (y-axis) for the 10 MND patients at the four cervical levels (indicated at the bottom right of each subfigure).

Statistically significant correlations between GM and WM areas were found at all levels in MND patients (Fig 4): C2-C3 (*r* = 0.848, p = 0.0019), C3-C4 (*r* = 0.851, p = 0.0018), C5-C6 (*r* = 0.906, p = 0.002), C7-T1 (*r* = 0.772, p = 0.0088).

In the ten HC correlations were weaker and not statistically significant at any level: C2-C3 (*r* = 0.307, p = 0.3879), C3-C4 (*r* = 0.488, p = 0.1529), C5-C6 (*r* = 0.606, p = 0.0635), C7-T1 (*r* = 0.105, p = 0.7728).

### Correlations of MRI metrics with clinical disability

There was no statistically significant correlation between GM and WM areas with the total ALSFRS-R, total DPR, and arm DPR.

## Discussion

Using the powerful PSIR imaging approach, we explored the involvement of cervical and upper thoracic cord GM and WM in MND patients. We found significant WM and GM atrophy in all patients with clinical involvement of the upper and/or lower extremities. In contrast, we did not detect GM or WM atrophy at any of the examined cord levels in the two bulbar onset ALS patients where motor symptoms remained restricted to the bulbar region. Interestingly, the three patients with PLS and PMA considered to have solely UMN or LMN involvement, demonstrated involvement of both GM and WM structures.

Regarding our hypothesis that MRI abnormalities would reflect the different phenotypes, our findings were conflicting among individual patients and across MND diagnoses. In the two bulbar onset ALS patients (#8 and #10) and the two FOSMN patients (#2 and #5), the MRI metrics were in line with the clinical findings. Patient #2 with a rapidly progressive disease course of FOSMN syndrome and fatal bulbar involvement, demonstrated solely LMN signs in the upper extremity and showed atrophy restricted to GM. The second FOSMN patient (#5) with widespread clinical involvement, both for anatomical regions and the upper and lower motor neuron, had the most severely reduced WM (total Z score -5.62) and GM (total Z score -4.41) areas among all patients examined. One of the two “typical” ALS patients (#3) with widespread clinical UMN and LMN involvement of the cervical and lumbar regions showed clear reductions in WM and GM areas on MRI. Patient #7 showed significant GM atrophy and a moderate WM atrophy at the C3-C4 and C5-C6 levels, but WM involvement was milder than that of GM. The FTD-ALS patient (#9) with bulbar and upper extremity involvement, also showed reductions in GM and WM areas. In contrast, the three patients with a clinical phenotype of solely UMN or LMN involvement, i.e. PLS (#6) and PMA (#1, #4), demonstrated concomitant reduction in GM and WM areas to an almost similar extent. This might indicate involvement of both UMN and LMN, even if clinically unaffected. These findings are supported by electrophysiological studies demonstrating involvement of the clinically unaffected UMN or LMN in a significant proportion of patients with PMA [3, 42] and PLS [43]. Of note, in our study MRI was the only method sensitive to the detection of this clinically silent motor neuron involvement, However, no electrophysiological studies to examine the upper motor neuron in the PMA patients were performed. In particular Transcranial Magnetic Stimulation (TMS) could serve this purpose [44, 45]. Furthermore, the EMG exam performed in the clinical routine did not cover all cord levels examined by PSIR imaging. On the other hand, PSIR might be detecting atrophy in areas of GM and WM that do not have a clinical correlate. For example we may be detecting atrophy in the anterior horn cells, but also in GM areas that do not correlate with LMN signs. Further studies are needed to systematically examine the added value of MRI atrophy measures to electrophysiological testing.

Another interesting finding of our study was the strong correlation between GM and WM area at all studied cord levels across the entire group (Fig 4), in spite of the heterogeneity of our MND cohort. This might point to a widespread neurodegenerative process that involves the entire motor system. The fact that the correlation was strong in MND patients and weaker in the ten HC, could be explained by a parallel process of atrophy of the GM and WM tissues in the MND patients that prevails over the natural inter-subject variability of the GM and WM areas as its extent increases. PSIR could be a viable modality to disentangle the spatiotemporal dynamics of focality and spread [46] among spinal cord GM and WM structures in MND.

To date, only two studies have assessed GM and WM spinal cord MRI metrics in ALS patients. In line with our findings, both studies demonstrated significant GM and WM atrophy in ALS [26, 27]. However, reported correlations between reductions in SC areas and disability are conflicting. The study by Rasoanandrianina et al. [27] including 10 patients with ALS, found weak correlations of TCA, but not GM area, with disability assessed by ALSFRS-R, and of upper motor neuron function with GM atrophy. The second study by Paquin et al. [26] included 29 patients and found similar correlations of GM area and TCA with clinical disability as measured by ALSFRS, both at baseline and after 1 year of follow up. Only GM area at baseline was predictive of clinical disability at 1-year follow up.

We did not find correlations between clinical scores and cervical spinal cord areas, even when using the arm ALSFRS subscore, which we assumed would clinically best correspond to the explored cervical cord region. This might be for several reasons. First, we applied the ALSFRS-R to the broader spectrum of MND, whereas it has only been validated for “typical” ALS. Second, besides the heterogeneous sample, we had a small sample size. In addition, the disadvantages of ALSFRS-R heterogeneity and the lack of correlations between clinical and imaging metrics across different MND study groups have been noted previously [35, 47]. The ALSFRS-R is used widely in clinical ALS trials to estimate the anatomical extent and clinical severity of motor involvement. However it does not differentiate upper from lower motor neuron symptoms, which is needed to segregate the different phenotypes within the MND spectrum.

An advantage of using PSIR is the ability to image lower cervical and upper thoracic cord levels. Previous approaches based on T2*-weighted contrasts report difficulties in imaging these anatomical cord segments due to technical challenges including susceptibility artifacts from motion, particularly swallowing and respiration [26]. PSIR is a T1-weighted inversion recovery sequence developed in cardiac imaging that is less sensitive to susceptibility and motion. Moreover, the short acquisition time of our PSIR protocol allowed us to repeat a scan if the image quality was suboptimal.

Taken together, our findings suggest that the detection of GM and WM atrophy patterns could aid in a better classification of distinct MND phenotypes, that might arise from different underlying pathophysiological mechanisms. Furthermore, determining the presence of GM and WM involvement might be clinically relevant, since MND patients with pure UMN or LMN phenotypes have a longer survival than “typical” ALS patients [46]. More work is needed to determine whether PSIR metrics can be meaningful at an individual patient level, but the cases presented in this work suggest that further exploration of this application is warranted.

### Limitations

The main limitation of this study is the relatively small sample size, thereby limiting general conclusions. However, the fact that we found significant reductions in GM and WM areas in a diverse population of MND diagnoses suggests a potential utility for this technique. A second limitation of this study is that the control group was not well matched for age and sex, however we addressed statistically by adjusting for those using regression analyses. A third limitation is the potential bias towards a slower progressing patient group, since rapidly progressing patients with severe respiratory symptoms were less likely to volunteer for a research MRI scan.

## Conclusions

In this study we performed a quantitative *in vivo* assessment of spinal cord GM and WM tissues in MND patients, using a short acquisition time PSIR protocol that could be easily added to any MRI clinical study. Detection of GM and WM atrophy in MND patients might help to better characterize clinical phenotypes and give insights into the focality and spread across the motor system in diseases of the MND spectrum. The subclinical detection of both GM and WM involvement in patients with PLS and PMA might be of prognostic value and should be further evaluated.

Future directions include exploring PSIR imaging in a wider variety of phenotypes in the spectrum of MND with larger sample sizes. Since PSIR imaging can be obtained throughout the cord, thoracic and even upper lumbar cord levels could be analyzed in the future with this technique.

## Supporting information

S1 Table Healthy controls data. Total cross-sectional area (TCA), gray matter area (GM) and white matter area (WM) for the ten healthy controls (HC) at the four cervical levels (C2-C3, C3-C4, C5-C6 and C7-T1) are reported. Group averages and standard deviations for the different areas are also reported.
(XLS)
